# Early Detection of Coronary Artery Disease by Micro-RNA Analysis in Asymptomatic Patients Stratified by Coronary CT Angiography

**DOI:** 10.3390/diagnostics10110875

**Published:** 2020-10-28

**Authors:** Andrew J. Patterson, Minwoo A. Song, David Choe, Daliao Xiao, Gary Foster, Lubo Zhang

**Affiliations:** 1 Lawrence D. Longo, MD Center for Perinatal Biology Department of Basic Sciences, Loma Linda University School of Medicine, Loma Linda, CA 92354, USA; minwoo0123@gmail.com (M.A.S.); Dxiao@llu.edu (D.X.); 2 Division of Cardiology Jerry L Pettis Memorial Veterans Hospital, Loma Linda, CA 92354, USA; dchoe@desertcard.com (D.C.); Gary@Fostertribe.net (G.F.)

**Keywords:** coronary artery disease, early detection, coronary CT angiography, Framingham Risk Score, micro RNA

## Abstract

Early detection of asymptomatic coronary artery disease (CAD) is essential but underdeveloped. The aim of this study was to assess micro-RNA (miRNA) expression profiles in patients with or without CAD as selected by coronary CT angiography (CTA) and stratified by risk of CAD as determined by Framingham Risk Score (FRS). In this pilot study, patients were divided into two groups based on the presence or absence of CAD. Disease status was determined by Coronary CTA by identification of atherosclerosis and/or calcified plaque in coronary arteries. There were 16 control subjects and 16 subjects with documented CAD. Groups were then subdivided based on FRS. Pathway-specific microarray profiling of 86 genes using miRNAs isolated from whole peripheral blood was analyzed. MiRNA were differentially expressed in patients with and without CAD and who were stratified on the basis of FRS with miRNA associated with endothelial function, cardiomyocyte protection and inflammatory response (hsa-miR-17-5p, hsa-miR-21-5p, hsa-miR-210-3p, hsa-miR-29b-3p, hsa-miR-7-5p and hsa-miR-99a-5p) consistently upregulated by greater than twofold in groups with CAD. The present study reveals that miRNA expression patterns in whole blood as selected on the basis of coronary CTA and risk scores vary significantly depending on the subject phenotype. Thus, profiling miRNA may improve early detection of CAD.

## 1. Introduction

Coronary artery disease (CAD) as a consequence of atherosclerosis is the leading contributor of mortality and morbidity facing the American and, increasingly, the global population [1,2]. Atherosclerosis is a byproduct of lifestyle in conjunction with familial genetic predisposition. Currently, CAD is primarily detected on the basis of clinical presentation, namely anginal symptoms. Angina often occurs late in the process of atherosclerosis as the atherosclerotic burden causes hemodynamic compromise of blood flow [3,4]. Stress testing or, depending on the severity and presentation, coronary angiogram are often indicated in the setting of ischemia [5]. Asymptomatic CAD is currently managed by mitigation of CAD risk factors. Framingham Risk Score (FRS) is the prototypical means of risk assessment [6,7]. FRS can be utilized as a preventive measure to minimize risk factors but does not predict disease in every patient. Patients can have high FRS without disease and low FRS with the disease [8,9]. Hence, a simple test that can detect early asymptomatic CAD is essential. 

Micro RNAs (miRNAs) are highly conserved non-coding RNAs; about 22 nucleotides in length, miRNAs are involved in differential regulation of genes expression through post-transcriptional degradation of mRNA and/or inhibition of translation [10,11]. MiRNAs are stable in extravascular fluids, often associated with lipid vesicles, lipoproteins (i.e., HDL) and other RNA binding proteins [12]. Studies in cardiovascular disease have found miRNA expression altered in circulating blood of patients with heart failure, ischemic cardiomyopathy and CAD [12,13]. Studies have identified several miRNAs that were dysregulated in a CAD cohort; however, most studies were conducted using a control population that did not undergo luminal screening [14,15,16]. MiRNA dysregulation is also observed in arrhythmia, cardiac remodeling, fibrosis, angiogenesis, cardiomyocyte survival and death in ischemic-reperfusion injury [17]. During a four-year follow-up of 1112 patients, selective miRNA obtained from peripheral blood reliably predicted cardiovascular death in acute coronary syndrome (ACS) patients [18]. Indeed, miRNAs is emerging as a possible tool to detect asymptomatic individuals with CAD.

Coronary CT angiography (CTA) is a modality that is able to detect early stages of vessel wall remodeling and calcification that are often missed in traditional angiograms [19,20,21,22]. Coronary CTA has been implemented in various clinical scenarios including acute coronary syndrome (ACS) risk stratification, emergency department chest pain protocols and outpatient clinical decision-making models [23,24]. The aim of this study was to assess miRNA expression patterns in subjects with and without CAD as determined by coronary CTA and further stratify on the basis of risk using FRS. We found differential regulation of miRNA involved in endothelial function, cardiomyocyte protection and inflammation in subjects with CAD, which were further magnified on the basis of 10 year cardiovascular risk prediction.

## 2. Methods

### 2.1. Patient Population

**Figure 1:**
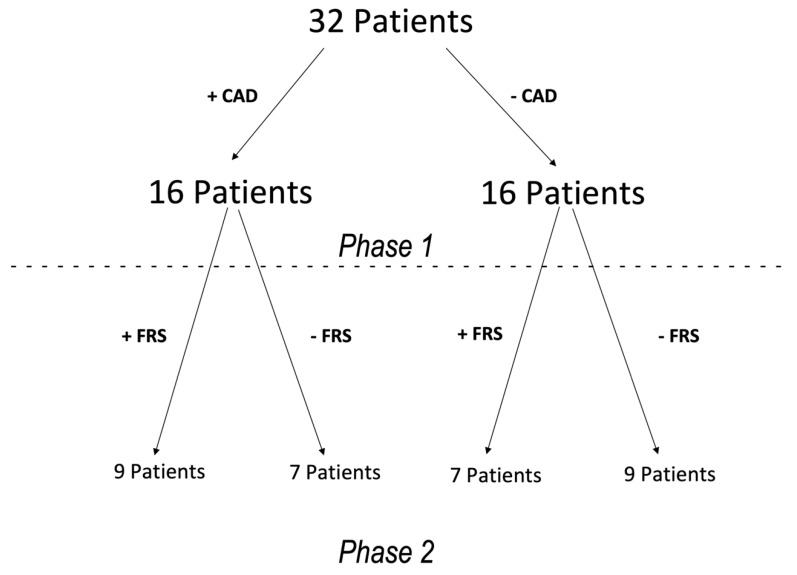
Diagram showing the 32 patients stratified on the basis of presence or absence of CAD (phase 1) and high or low FRS (Phase 2). CAD—Coronary Artery Disease. FRS—Framingham Risk Score.

This pilot study was performed at a single center. Prior to patient recruitment, the protocol was approved through the Loma Linda VA Institutional Review Board, and the study was conducted in accordance with the Declaration of Helsinki. All subjects signed an informed consent to be included in this study. Asymptomatic patients with known history or suspicion of CAD were recruited along with volunteer subjects without history or clinical suspicion for coronary artery disease between 2007 and 2012 to undergo coronary CTA. During the initial phase, subjects were divided based on the presence or absence of CAD (Figure 1). In the second phase, subjects were subdivided on the basis of FRS (Figure 1). Low FRS was classified as 10 year event rate of <5%, and high FRS was classified as a 10 year event rate of >18% [6,25]. General exclusion criteria included subjects with contraindication for coronary CTA, elevated ESR/CRP and FRS between 6 and 17%. Contraindications for undergoing coronary CTA were atrial fibrillation and GFR <40 mL/min. In addition, patients with unstable angina, known history of thrombocytopenia, or severe hepatic or renal dysfunction, as well as evidence for malignant disease were excluded. 

### 2.2. Coronary CTA

**Figure 2:**
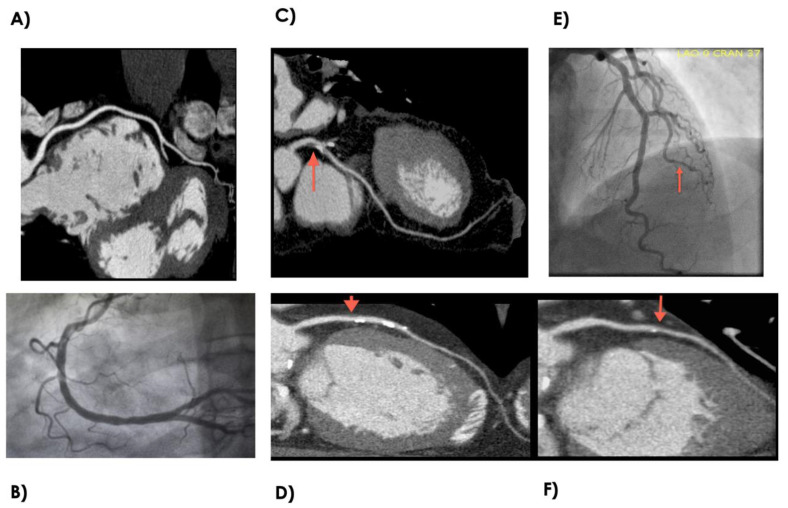
Example of normal and disease coronary anatomy as depicted by coronary CTA and coronary angiogram. (**A**) Normal right coronary artery by coronary CTA. (**B**) Normal right coronary artery by coronary angiogram. (**C**) Focal Mixed calcified atherosclerotic plaque in the proximal left anterior descending artery as depicted by coronary CTA. (**D**) Sequential soft lipid and calcified plaque in the mid-to-proximal left anterior decending artery as depicted by coronary CTA. (**E**) Luminal irregularities involving the 2nd diagonal branch as depicted by coronary angiogram. (**F**) Focal luminal remodeling with mix soft lipid plaque and spotty calcifications. CTA—Computed Tomography Angiography.

All coronary CTA scans were performed in a Siemens dual-source 64-multidetector SOMATOM CT. Preferred sinus rate was less than 65 bpm, but patients were scanned whether or not patient’s heart rate was less than 65 bpm. One or more doses of metoprolol 5mg IV were given up to 25 mg to slow the heart rate. For coronary artery calcification measurements, an ECG-gated non-contrast scan was performed with 2.5 mm thickness. Agaston score was calculated using a standard measurement method (26). For plaque visualization, a scout CT was performed with 10–20 mL of contrast (Omnipaque, GE Healthcare, Buckinhamshire, United Kingdom) to calculate optimal contrast administration timing, with contrast being detected in the proximal ascending aorta. Contrast enhancement was performed with 80 mL of contrast (Omnipaque, GE, Buckinhamshire, United Kingdom) in a triple-phase injection protocol: 60 mL of contrast was followed by 40 mL of a 50:50 mixture of contrast and saline, then finished with a 50 mL saline flush. Helical CT was performed with contrast from 20 mm above the left main artery to 20 mm below the inferior wall using retrospective ECG-gating. Coronary CTA images were evaluated for the presence of coronary artery disease by an experienced cardiologist. Coronary CTA determined disease status through the identification of calcium or plaque in the coronary arteries. Patients with zero coronary calcium and no identifiable plaque were classified as controls. The CAD group had a minimum of one defined coronary plaque within a minimum of one epicardial coronary artery. See Figure 2 for examples of coronary CTA and coronary angiogram.

### 2.3. MiRNA Isolation and Purification 

Whole peripheral blood samples (2 mL) were washed and processed for total RNA extraction using the PAXgene Blood RNA Isolation kit (Qiagen, Valencia, CA, USA). Samples were stored at −70 °C until PCR was performed. Prior to PCR, the quality of RNA was documented for quality and fidelity. Total RNA was then diluted 1:10, then 5 μL was reverse-transcribed using the TaqMan microRNA Reverse Transcription Kit (ABI) to create cDNA. Qiagen Human Cardiovascular Disease miRNA PCR Array MIHS-113Z was used with 3 μL aliquots of cDNA, miScript Universal Primer, and SYBR Green PCR master mix in a real-time PCR cycler.

### 2.4. MiRNA Microarray

Total RNA preparation was reverse-transcribed using TaqMan microRNA Reverse Transcription from miScript miRNA selective buffering technique to determine the miRNA expression pattern. We performed pathway-specific microarray profiling on 84 miRNAs known to exhibit altered expression patterns in cardiovascular disease. A list of miRNA candidate genes was included in Table 1. Microarray results were normalized using 6 snoRNA/snRNA miScript controls (Table 1). These small RNAs have been verified to have relatively stable expression levels across multiple tissue and cell types. ΔCT value for each mature miRNA profiled in the plate was calculated using the formula ΔCT = C_T_^miRNA^ – AVG C_T_^SN1/2/3/4/5/6^. ∆∆C_T_ for each miRNA across 2 miScript miRNA PCR Arrays or 2 samples was calculated using the formula: ∆∆C_T_ = ∆C_T_ (sample 2) – ∆C_T_ (sample 1), where sample 1 is the control sample and sample 2 is the experimental sample. Fold-change for each gene from sample 1 to sample 2 is calculate as 2^(−∆∆CT)^.

### 2.5. Statistical Analyses

Demographic and clinical variables were compared between groups using the *t*-test for continuous variables and the Chi-square test or Fisher’s exact test for categorical variables. Raw C_T_ values were analyzed using the miScript miRNA PCR Array Data Analysis Tool, which automatically interprets the PCR array controls, performs quantification using the ∆∆C_T_ method listed above, and presents the results in volcano plot and heat map format. The data analysis portal can be found at https://dataanalysis.qiagen.com/mirna/arrayanalysis.php. The volcano plot displays the relationship between fold-change and significance between the two groups. For significance, greater than twofold increase or decrease with *p* values < 0.05 were used for cut-offs.

## 3. Results

### 3.1. Study Population Characteristics 

A total of 32 subjects, 16 control and 16 CAD, were selected for the study (Figure 1). The reference healthy control group were subjects without CAD and with low FRS. Group 1 were subjects with CAD and high FRS. Group 2 were subjects without CAD but with high FRS. Group 3 were subjects with CAD but with low FRS. Coronary CTA determined disease status through the identification of calcium or plaque in the coronary arteries. Figure 2 shows examples of normal and diseased coronary arteries as demonstrated with coronary CTA and coronary angiogram. The clinical characteristics of each group are summarized in Table 2. There were significant differences for age, gender, coronary artery calcium score (CACS), dyslipidemia between healthy controls and disease cohorts. Furthermore, there were differences for patients on anti-platelet, beta-blockers and ACEI/ARB therapy between patients with CAD and healthy controls.

### 3.2. miRNA Profile in Subjects with CAD Versus Healthy Controls

**Figure 3:**
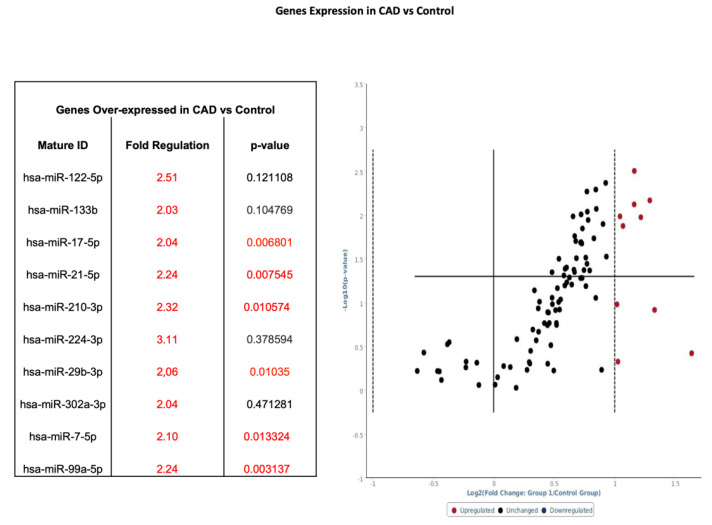
Volcano plot with corresponding table showing the comparison of miRNA assessed by microarray (84 genes) isolated from patients with CAD (*n* = 16) versus healthy controls (*n* = 16). Designation of significance (RED) was given to samples with fold change >2. Samples with *p* values < 0.05 and fold change > 2 are found in the upper right quadrant. CAD—Coronary Artery Disease.

Whole blood samples in CAD patients (*n* = 16) were analyzed by using pathway-specific cardiovascular disease microarray gene chip and compared to healthy controls (*n* = 16) (Figure 1 phase 1). Among the 86 genes surveyed, 10 genes had a twofold-to-threefold increase from baseline and 6 of those genes showed statistical significance when comparing CAD to control (Figure 3). 

### 3.3. Group 1: miRNA Profile in Subjects with CAD and High FRS

**Figure 4:**
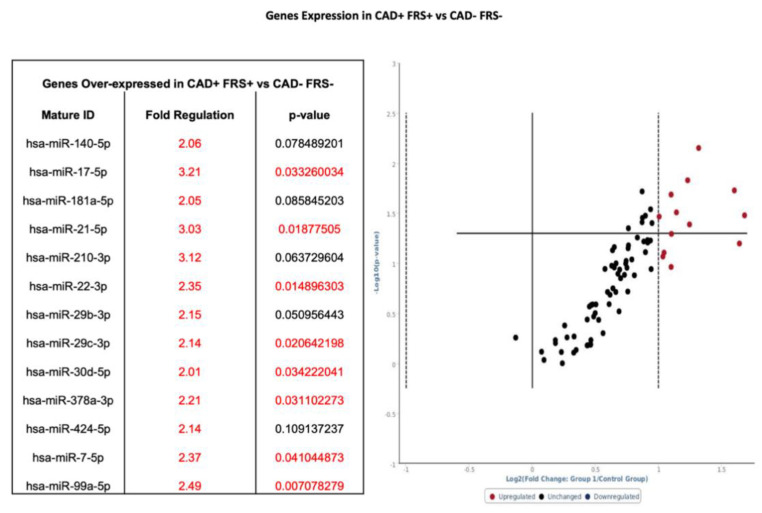
Volcano plot with corresponding table showing the comparison of miRNA assessed by microarray (84 genes) isolated from patients with CAD and FRS > 18 (*n* = 9) versus heathy controls without CAD and FRS < 5 (*n* = 9). Designation of significance (RED) was given to samples with fold change >2. Samples with *p* values < 0.05 and fold change >2 are found in the upper right quadrant. CAD—Coronary Artery Disease. FRS—Framingham Risk Score.

Patients were subdivided into those with CAD and FRS > 18 (*n* = 9) and those without CAD and FRS < 6 (*n* = 9) (Figure 1—Phase 2). Whole blood samples in CAD patients with high FRS (*n* = 9) were analyzed by using pathway-specific cardiovascular disease microarray gene chip. Similar to total sampling, 13 genes had twofold-to-threefold or greater increase from baseline expression, and eight of those genes showed statistical significance when compared to control samples (Figure 4). Of note, hsa-miR-17-5p, hsa-miR-21-5p, hsa-miR-210-3p, hsa-miR-29b-3p, hsa-miR-7-5p, and hsa-miR-99a-5p were increased by at least twofold, whether separated on the basis of CAD (see Figure 3) or further stratified by FRS (see Figure 4).

### 3.4. Group 2: miRNA Profile in Subjects without CAD and High FRS

**Figure 5:**
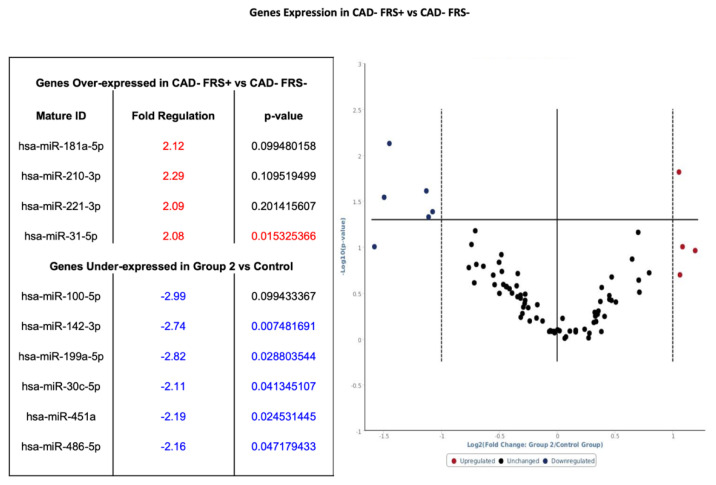
Volcano plot with corresponding table showing the comparison of miRNA assessed by microarray (84 genes) isolated from patients without CAD but FRS > 18 (*n* = 7) versus heathy controls without CAD and FRS < 5 (*n* = 9). Designation of significance (RED) was given to samples with fold increase >2 and (Blue) was given to samples with fold decrease >2. Samples with *p* values < 0.05 and fold change >2 are found in the upper right quadrant and upper left quadrant. CAD—Coronary Artery Disease. FRS—Framingham Risk Score.

Within the seven patients without CAD and high FRS (Figure 1—Phase 2), four genes had greater than a twofold increase from baseline expression and six genes had a twofold or more decrease from baseline. Among the four genes overexpressed, one showed statistical significance, and among six genes under-expressed, five exhibited statistical significance when compared to the control (see Figure 5).

### 3.5. Group 3: miRNA Profile in Subjects with CAD and Low FRS

**Figure 6:**
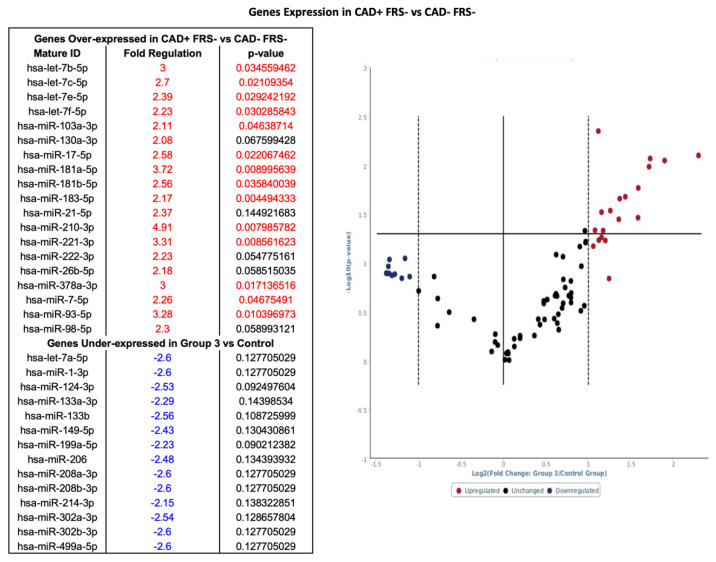
Volcano plot with corresponding table showing the comparison of miRNA assessed by microarray (84 genes) isolated from patients with CAD and FRS < 5 (*n* = 7) versus heathy controls without CAD and FRS < 5 (*n* = 9). Designation of significance (RED) was given to samples with fold increase >2 and (Blue) was given to samples with fold decrease >2. Samples with *p* values < 0.05 and Figure 2. are found in the upper right quadrant and upper left quadrant. CAD—Coronary Artery Disease. FRS—Framingham Risk Score.

Nineteen genes had a greater than twofold increase in expression in patients with CAD with low FRS as compared to patients without CAD and low FRS (see Figure 1). Among 19 genes overexpressed, 14 were statistically significant (see Figure 6). In addition, 14 genes exhibited a twofold or more decrease in expression; however, genes that were twofold or more decreased did not have significant *p* values (Figure 6). Of note, hsa-miR-17-5p, hsa-miR-210-3p and hsa-miR-7-5 were upregulated in all patients with CAD. 

### 3.6. Clustergram of miRNA Profiles in Subdivided Groups 

**Figure 7:**
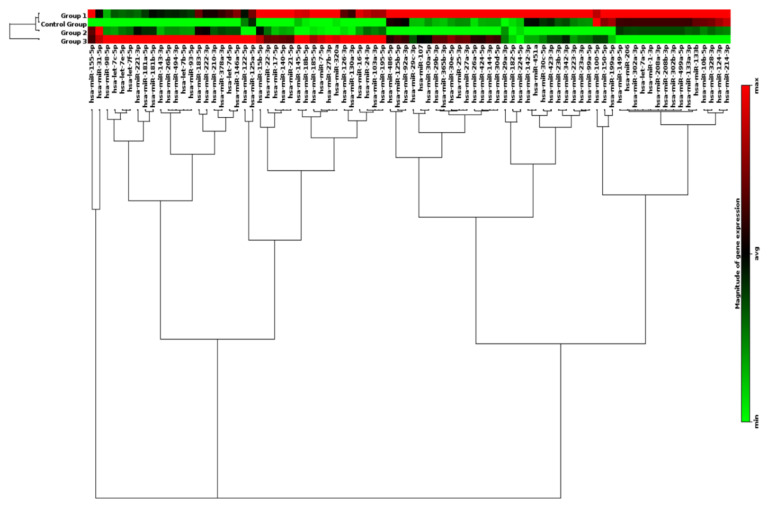
Profile of circulating miRNAs in CAD patients. MiRNA Heat Map illustrates differential expression between groups stratified by the presence or absence of CAD and risk assessment by FRS. Color intensity is scaled within each row such that the highest expression value corresponds to bright red, and lowest to bright green. CAD—Coronary Artery Disease. FRS—Framingham Risk Score.

The clustergram performs non-supervised hierarchical clustering of the entire dataset to display a heat map with dendrograms indicating co-regulated genes across individual, or in this case grouped, samples. Overall, 86 genes were surveyed, and distinct expression patterns were displayed. As shown in Figure 7, differential expression patterns of groups of miRNAs clearly separated patients with CAD (Groups 1 and 3) from those without CAD (Control and Group 2). In addition, within the patients who have CAD, differential expression profiles of a group of 15 miRNAs further distinguished CAD patients with high FRS (Group 1) from those with low FRS (Group 3). However, there were no clear differences in miRNA profiles examined between low (Control Group) and high (Group 2) FRS in patients who have no CAD. 

## 4. Discussion

To our knowledge, this is the first study to utilize coronary CTA to stratify subjects with or without CAD for microarray analysis of miRNAs in whole blood. This pilot study presents preliminary evidence consistent with previous studies that CAD alters the expression patterns for a selected panel of epigenetic regulatory nucleotides.^14^ We present preliminary evidence that suggests that populations stratified based on FRS further magnified differences in miRNA levels in patients with and without CAD. Consequently, our data add to existing knowledge that miRNA profiling in blood may allow for early identification of CAD in asymptomatic patients. 

Our study adds additional insight into the expression patterns of miRNA in patients with CAD. Previous studies have published data examining the miRNA expression profile in whole blood, plasma, and peripheral blood mononuclear cell (PBMC) using angiographic techniques as a means of confirming patients with CAD. We believe that our approach of using Coronary CT angiography affords the advantage of noninvasive screening, better discrimination between control and disease groups through visualization of coronary vessel walls, and thus allowing early recognition of vessel remodeling and calcium scoring, which allows for risk stratifying low-risk asymptomatic patients [25,26]. Figure 2 shows examples of subtle disease difficult to see on angiogram and the improved view of coronary vessel wall, allowing for better determination of presence of disease. In addition, the expression patterns of miRNA between angiographically positive and negative subjects can potentially differentiate between negative-to-mild CAD and moderate-to-severe CAD or high-risk coronary plaque from stable plaques. The application of CTA and miRNA allows for greater confidence in discriminating CAD patterns of disease from normal subjects. The limitation is that not every patient is able to undergo coronary CTA owing to requirements for baseline renal function and need for adequate heart rate control. 

Biochemically, our data suggest that when patients were segregated by the presence or absence of CAD or if further stratified on the basis of low or high FRS, a unique miRNA distinction is seen. Figure 3 and Figure 4 show several miRNAs that are significantly upregulated in patients with CAD and/or high Framingham Risk Score. Increased expression of miR-17, -21, -210, -22, and -378 are of interest since they have been documented in conferring cardiomyocyte survival after ischemia-reperfusion (IR) injury. In larger studies looking for markers to predict risk of cardiovascular death, miRNA-210 was shown to be associated with risk of cardiovascular death (18). Upregulation of miR-17 and -21 leads to suppression of ischemia-induced increase in expression of PTEN and FasL [27,28]. MiR-17 directly targets TMP-3 and indirectly inhibits PTEN, promoting survival by ameliorating heart failure and reducing infarct size [28]. MiR-22 and miR-378 target pro-apoptotic genes related to Bax and caspase-3, and markedly reduce IR injury in murine models [29,30]. Elevated expression of miR-17, -21, -210, -22, and -378 suggests a compensatory mechanism of cardiomyocytes to minimize apoptosis under a perfusion deficit environment. It is difficult to explain as these are preliminary data; however, the miRNA pattern suggests dynamic regulation under prolonged ischemic conditions. Although it is important to understand the underlying mechanism of individual miRNA, the pattern of expression of groups of miRNAs may hold the key to identifying disease presence and progression in CAD. The expression of miR-99-5p was significantly increased in CAD groups compared to non-CAD groups. Studies conducted in human aortic smooth muscle cells and mice models show that miR-99-5p uniquely plays a role in preventing atherosclerosis via inhibition of HOX1A expression [31]. Additionally, miR-29b-3p appears to regulate vascular smooth muscle calcification through a MMP2 regulation [32]. Our initial experience using clustergrams demonstrated clear distinctions between the CAD and healthy controls in the pattern of miRNA expression. As seen in Figure 7, miR-15b through miR-195 show clear upregulation in Groups 1 and 3 as compared to control and Group 2. Groups 1 and 3 are patients with CAD, suggesting a pattern of regulation novel to patients with CAD. The downstream implication of these findings is unclear. Future studies validating these findings are warranted. The specific pattern demonstrated in the present study may potentially guide larger studies examining early detection of CAD and evaluate the risk of an acute coronary syndrome in patients with high FRS.

Subjects with elevated risk of coronary disease yet who did not have evidence of major epicardial disease are represented in Group 2 (Figure 5). The miRNA expression profile was unique and may suggest a form of ischemic preconditioning (IP). Studies have implicated several miRNA involving cardiomyocyte survival in IR injury and protection from acute coronary syndrome [33,34,35] Of the miRNA differentially regulated in Group 2 (see Figure 6), miR-199a appears to have an important role since it regulates hypoxia-inducible factor-1α (HIF-1α) and proliferator-activated receptor (PPAR) δ in favor of PPAR γ. HIF-1α is considered as the central regulator promoting preconditioning by hypoxia-triggered pathways [34]. MiR-199 directly targets Sirtuin (Sirt) 1, which is responsible for stabilizing HIF-1α [36]. Therefore, downregulation of miR-199a by threefold may increase HIF-1α availability. miR-199a represses PPAR δ in favor of PPAR γ, which shifts the dependency of the heart from fatty acid to glucose as a major energy expenditure [37]. PPAR δ is responsible for fatty acid utilization by the adult heart as a primary source of energy. The metabolic shift to PPAR γ may be detrimental due to increased demands for oxygen in already stressed microtissue environment. Other pathways implicated by downregulated nucleotides in Group 2 are involved in protection against reactive oxygen species, reduction of the apoptotic pathway, and inhibition of fibroblast activity [38,39,40]. The dysregulation of the observed miRNAs provides a unique view of molecular pathways governing tissue-specific responses to chronic disease states known to be associated with CAD. Whether these findings represent a protective or long-term detrimental adaptation is unclear and warrants further research and phenotypic characterization.

Group 3 represents subjects who were at lower risk of coronary disease but ironically displayed evidence of epicardial coronary disease. This phenotype is quite perplexing clinically as this group of individuals often are well controlled medically but still present with CAD. The discordance between risk factors and presence of CAD presents unique insight into pathogenesis of CAD and highlights the current limitation of risk factor scores to predict CAD risk. Interestingly, miR-17, -7, and -378a elevated in both Groups 1 and 3. MiR-378a inhibits apoptosis cascade by inhibiting caspase-3 activity [30], while miR-17 and -7 are important in the regulation of inflammation and endothelial function. These findings could potentially indicate that miRNAs may be markers of ischemic stress secondary to CAD. Other elevated miRNAs, miR-93, -210 and -181b, have been implicated in cardiomyocytes protection after IR injury [41,42,43] and in the setting of hypoxic stress [44]. One question remains regarding the profile of patients with CAD and high FRS compared to those with CAD and concomitant familiar dyslipidemias and to what extent small noncoding nucleotides regulate phenotypic expression. Additionally, it is unclear if plaque characteristics such as calcified, fibrofatty, fibrous or necrotic core are influenced by or influences miRNA expression patterns. How this differs in plaque with clinically significant CT fractional flow reserve (FFR), a measure of physiological stenosis and estimates of total plaque burden is unknown and intriguing. Future studies examining subjects selected on the basis of the predominated anatomical features, positive or negative CT-FFR or separated on the basis of quartiles based on total plaque burden may offer significant insight.

There are various limitations to the study. First, the study was conducted in a single center and was not powered for large-scale biomarker evaluation, but instead was conceived as an initial experiment to report initial concept exploring differences in miRNA expression utilizing coronary CTA and FRS for risk stratification. Coronary CTA use in a broader scale is limited by requirements of appropriate renal function and need for adequate heart rate control, which can influence population selection heterogeneity. Therefore, the results should be interpreted in light of these limitations. The patient cohort had significant differences in the use of antiplatelets, beta-blockers and ACEI/ARBs and it is unclear the effect these drugs may have on circulating miRNA. Additionally, there are differences in terms of age, gender and diagnosis of dyslipidemia. The larger skew towards males is reflective of the veteran population where the study was conducted. Elucidating the miRNA profile in a predominately female cohort may yield differences in miRNA profile in whole blood. Although there was a significant difference in patients’ diagnosis with dyslipidemia, there were no difference in lipid profiles at the time of the study. These findings reflect our best efforts to obtain a disease-free population that were absent of epicardial coronary arterial disease with an overall low FRS. Validation of candidate miRNA in a larger sample size in the general population is the natural next step. Patients were not stratified based on clinically significant CAD (<50% stenosis) versus non-stenotic disease and/or presence of soft lipid plaque versus calcified lesions, which may yield a different pattern of expression. Additionally, whole blood samples were not separated into their representative constitutes (Plasma and PBMC). We initially attempted to extract miRNAs from plasma but obtained low yields. We then utilized whole blood samples, which only showed global, not specific, compartmental regulation. Finally, the mechanistic pathways governing the end product of miRNA expression were not elucidated in this model. As this was the initial experience and proof of concept, further studies utilizing animal models designed to interrogate specific genes or therapeutic knockouts of miRNA are future steps which we believe will provide additional regulatory insight. 

Coronary CTA allows evaluation of the vessel wall, providing a more reliable method to stratify subjects with and without CAD, a notable improvement on prior studies relying on stratification limited to history and invasive arteriography. In this initial experience utilizing FRS, we discovered unique miRNA patterns that may help to identify asymptomatic patients with CAD. The use of miRNA as a tool to identify individuals with CAD continues to show promise and larger studies examining the miRNA patterns in CAD are warranted. Although there remain barriers to the implementation of miRNA-based studies in clinical practice, the potential is greatly illustrated by this pilot study.

## Figures and Tables

**Table 1:** List of MiRNA Array Panel.

Gene Symbol	Gene Symbol	Gene Symbol	Gene Symbol	Gene Symbol	Internal Controls
hsa-let-7a-5p	hsa-miR-130a-3p	hsa-miR-182-5p	hsa-miR-23a-3p	hsa-miR-31-5p	SNORD61
hsa-let-7b-5p	hsa-miR-133a-3p	hsa-miR-183-5p	hsa-miR-23b-3p	hsa-miR-320a	SNORD68
hsa-let-7c-5p	hsa-miR-133b	hsa-miR-185-5p	hsa-miR-24-3p	hsa-miR-328-3p	SNORD72
hsa-let-7d-5p	hsa-miR-140-5p	hsa-miR-18b-5p	hsa-miR-25-3p	hsa-miR-342-3p	SNORD95
hsa-let-7e-5p	hsa-miR-142-3p	hsa-miR-195-5p	hsa-miR-26a-5p	hsa-miR-365b-3p	SNORD96A
hsa-let-7f-5p	hsa-miR-143-3p	hsa-miR-199a-5p	hsa-miR-26b-5p	hsa-miR-378a-3p	RNU6-6P
hsa-miR-1-3p	hsa-miR-144-3p	hsa-miR-206	hsa-miR-27a-3p	hsa-miR-423-3p	
hsa-miR-100-5p	hsa-miR-145-5p	hsa-miR-208a-3p	hsa-miR-27b-3p	hsa-miR-424-5p	
hsa-miR-103a-3p	hsa-miR-146a-5p	hsa-miR-208b-3p	hsa-miR-29a-3p	hsa-miR-451a	
hsa-miR-107	hsa-miR-149-5p	hsa-miR-21-5p	hsa-miR-29b-3p	hsa-miR-486-5p	
hsa-miR-10b-5p	hsa-miR-150-5p	hsa-miR-210-3p	hsa-miR-29c-3p	hsa-miR-494-3p	
hsa-miR-122-5p	hsa-miR-155-5p	hsa-miR-214-3p	hsa-miR-302a-3p	hsa-miR-499a-5p	
hsa-miR-124-3p	hsa-miR-15b-5p	hsa-miR-22-3p	hsa-miR-302b-3p	hsa-miR-7-5p	
hsa-miR-125a-5p	hsa-miR-16-5p	hsa-miR-221-3p	hsa-miR-30a-5p	hsa-miR-92a-3p	
hsa-miR-125b-5p	hsa-miR-17-5p	hsa-miR-222-3p	hsa-miR-30c-5p	hsa-miR-93-5p	
hsa-miR-126-3p	hsa-miR-181a-5p	hsa-miR-223-3p	hsa-miR-30d-5p	hsa-miR-98-5p	
	hsa-miR-181b-5p	hsa-miR-224-5p	hsa-miR-30e-5p	hsa-miR-99a-5p	

**Table 2:** Clinical Characteristics of Patients with CAD and Healthy Controls.

Characteristics	Patients with CAD (*n* = 16)	Healthy Controls (*n* = 16)	*p* Value
Age (years)	61.5 ± 11.6	46.1 ± 10.8	<0.001
Male Gender (X)	16	12	0.013
Framingham Score	14.2 ± 7.7	8.1 ± 7.9	0.016
CACS	518.9 ± 631.6	0	0.001
Systolic Blood Pressure	129.8 ± 13.9	122.5 ± 22.1	0.326
Type 2 Diabetes (n)	5 (29%)	2 (12.5%)	0.124
Dyslipidemia (n)	11 (65%)	5 (31%)	0.029
CHF (n)	1 (6%)	0	0.17
Active Smoker (n)	7 (41%)	9 (56%)	0.283
Total Cholesterol	173.2 ± 42.2	193.8 ± 45.1	0.098
LDL-Cholesterol	98.6 ± 40.5	116.4 ± 35.4	0.169
HDL-Cholesterol	45.9 ± 15.1	37.4 ± 17.7	0.075
		Medications (n)	
Antiplatelet	8 (47%)	1 (6%)	0.004
β-Blockers	8 (47%)	2 (12.5%)	0.016
Stain	9 (53%)	8 (50%)	0.435
ACE or ARB	9 (53%)	3 (19%)	0.045
Calcium Channel Blocker	4 (24%)	2 (12.5%)	0.214

CACS: coronary artery calcium scoring.
